# The Impact of Spatial Incongruence on an Auditory-Visual Illusion

**DOI:** 10.1371/journal.pone.0006450

**Published:** 2009-07-31

**Authors:** Hamish Innes-Brown, David Crewther

**Affiliations:** Brain Sciences Institute, Swinburne University, Melbourne Australia, Hawthorn, Victoria, Australia; Center for Genomic Regulation, Spain

## Abstract

**Background:**

The sound-induced flash illusion is an auditory-visual illusion – when a single flash is presented along with two or more beeps, observers report seeing two or more flashes. Previous research has shown that the illusion gradually disappears as the temporal delay between auditory and visual stimuli increases, suggesting that the illusion is consistent with existing temporal rules of neural activation in the superior colliculus to multisensory stimuli. However little is known about the effect of spatial incongruence, and whether the illusion follows the corresponding spatial rule. If the illusion occurs less strongly when auditory and visual stimuli are separated, then integrative processes supporting the illusion must be strongly dependant on spatial congruence. In this case, the illusion would be consistent with both the spatial and temporal rules describing response properties of multisensory neurons in the superior colliculus.

**Methodology/Principal Findings:**

The main aim of this study was to investigate the importance of spatial congruence in the flash-beep illusion. Selected combinations of one to four short flashes and zero to four short 3.5 KHz tones were presented. Observers were asked to count the number of flashes they saw. After replication of the basic illusion using centrally-presented stimuli, the auditory and visual components of the illusion stimuli were presented either both 10 degrees to the left or right of fixation (spatially congruent) or on opposite (spatially incongruent) sides, for a total separation of 20 degrees.

**Conclusions/Significance:**

The sound-induced flash fission illusion was successfully replicated. However, when the sources of the auditory and visual stimuli were spatially separated, perception of the illusion was unaffected, suggesting that the “spatial rule” does not extend to describing behavioural responses in this illusion. We also find no evidence for an associated “fusion” illusion reportedly occurring when multiple flashes are accompanied by a single beep.

## Introduction

In cases where multiple senses provide congruent information concerning the same external event, multisensory integration can result in perceptual advantages. For instance, the detection of weak visual stimuli is enhanced with a concurrent auditory stimulus [1], and speech perception is improved when video of the moving lips is available [2], [3]. However, if an experimenter arranges incongruent information to be presented to each sensory modality, the senses can also interfere with each other, causing altered or illusory percepts. Perhaps the best-known example of this phenomenon is the McGurk-MacDonald illusion [4], [5], where vision alters the way a speech sound is perceived. When the audio for “ba” is presented along with video of an actor pronouncing “ga”, the consonant half-way between (“da”) is perceived. In the majority of these illusions, visual information tends to dominate in the spatial domain.

In rarer cases, particularly where temporal information is involved, audition may dominate visual perception. The sound-induced flash illusion [6] is one such case. In this experiment, a varying number of flashes were presented along with a varying number of short beeps. When a single flash was presented along with two or more beeps, observers often reported seeing two or more flashes. In an event-related potential (ERP) study of the same illusion [7], the auditory stimulus was found to modify the flash visual evoked potential (VEP). The authors proposed that the illusion is perceptual – that the second auditory stimulus caused the illusory perception of a second flash. Other evidence from EEG [8], [9] and MEG [10] has shown neurophysiological correlates of the illusion in primary visual areas, thus giving weight to the suggestion that the illusion is perceptual rather than a result of response biases. fMRI studies [11], [12], [13] have also found increased functional activity associated with the illusion in V1 as well as the superior temporal sulcus (STS) and superior colliculus (SC) [11]. Both these structures have previously been associated with the integration of auditory and visual information [14], [15], [16]. A corresponding flash-fusion illusion has also been reported, where a single beep causes fusion of a double flash stimulus [17], [18]. Few other behavioural investigations of the illusion have been performed (although see McCormick & Mamassian [19] for a recent exception).

Studies using single-cell electrophysiology [20] have found that certain neurons in the superior colliculus (SC) are responsive to stimulation in more than one sensory modality. Interestingly, many of these cells show a response gradient where visual and auditory stimuli occurring in close spatial and temporal proximity cause cells to respond more strongly than would be expected by simple addition of the individual responses to each uni-sensory stimulus. These properties have been described by three rules [20]. The *spatial rule* states that only stimuli from different modalities that are in close spatial proximity are integrated and produce response enhancement. This reflects the manner in which maps of space across different modalities are aligned in the SC. The *temporal rule* states that multisensory stimuli are more likely to be integrated when they occur at similar times, reflecting the way in which maximal multisensory enhancement occurs when the responses to each unisensory stimuli are at their peaks [21]. The *inverse effectiveness* rule describes how individually weak unisensory stimuli combine to produce larger neural responses than would be expected by the simple addition of each individual response. Together, these rules have provided a conceptual framework for mapping the behavioural consequences of multisensory integration to possible underlying physiological properties. These physiological data seem to correlate well with human behavioural performance, which also shows spatial [22] and temporal [1] response gradients. The sound-induced flash illusion is known to gradually vanish with increasing temporal incongruence [23], and thus is consistent with the temporal rule, however little is known about how spatial incongruence might affect the illusion.

In addition to structures such as the SC, multisensory integration can also occur rapidly via direct cortico-cortical pathways (see Driver and Noesselt [24] for a review). Direct projections to V1 from the core and belt regions of the auditory cortex as well as upper banks of the superior temporal sulcus (STS) have been found in monkeys [25], and these connections appear to target mainly the peripheral region of the visual field [25]. Somatosensory-responsive regions within human auditory cortex have been found using fMRI [26] however these early interactions do not appear sensitive to spatial congruence [27].

In the environment, auditory and visual signals from a common event or object are often spatially and temporally congruent, and the mechanisms underlying multisensory integration seem bound by corresponding rules, which are in turn reflective of the underlying properties of multisensory neurons. The aim of this study is to investigate the effect of spatial incongruence in the sound-induced flash illusion. If the illusion occurs less strongly when auditory and visual stimuli are separated, then integration supporting the illusion must be strongly dependent on spatial congruence. In this case the illusion would be consistent with both the spatial and temporal rules describing response properties of multisensory neurons in the SC. Conversely, if perception of the illusion is unaffected, and multisensory integration occurs regardless of the spatial congruence of the auditory and visual stimuli, then the “spatial rule” may not apply to this illusion, suggesting that multisensory integration could be subserved by parts of the brain not known to be governed by these rules.

## Methods

### Ethics Statement

The study conforms to The Code of Ethics of the World Medical Association (Declaration of Helsinki), and was approved by the Swinburne University Human Research Ethics Committee. All participants gave informed consent.

### Participants

Nine healthy adults (five male, *M = *26.9 years, *SD* = 4.2) participated in the study after providing written informed consent. Each participant reported normal or corrected-to-normal vision and hearing.

### Stimuli

In experiment one, the stimulus configuration followed the original report[23] as closely as possible. The experiment was conducted in a quiet room with an average background sound level of 39 dB (A-weighted). Brief flashes were presented on a cathode-ray tube (CRT) computer monitor. Short beeps were presented along with the flashes from two small speakers placed centrally under the monitor. The centre of the speaker was 8 degrees below the visual stimulus.

The visual stimulus consisted of a white disk on a black background flashed from one to four times. The disk subtended 2° of visual field and was located 5° below a small central fixation cross. The refresh rate of the CRT monitor was set to 60 Hz (the refresh period was thus 16.7 ms), and each flash was set to display for one refresh period of the monitor. In order to determine the exact flash duration the persistence of the CRT phosphors was measured using a photodiode and oscilloscope. The flash duration was 1.3 ms (full width at half maximum - FWHM). In trials where more than one flash was presented, the next flashes followed after three blank refresh periods. The between-flash onset asynchrony was thus 67 ms.

**Figure 1:**
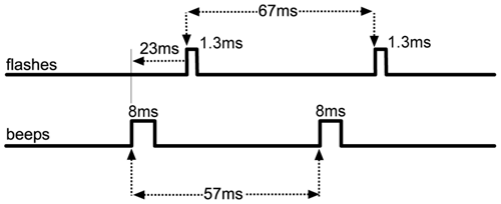
Design. Stimulus timing for flashes and beeps. A 2-flash 2-beep (2F2B) trial is shown here.

The beep was a 3500 Hz, 85 dB (A-weighted) sine wave of 8 ms duration (3 ms rise/fall times). On one-flash trials, zero to four beeps were presented. On multi-flash trials, either zero or one beep was presented. For relative timing information see Fig. 1. The first beep was always presented 23 ms prior to the first flash, and in trials where more than one beep was presented, the between-beep asynchrony was 57 ms. The stimulus combinations will be referred to using abbreviations for the number of flashes (F) followed by the number of beeps (B), such that “1F0B” refers to a single flash with no beeps.

In the second experiment, the basic physical characteristics of the stimuli were identical to those used in experiment one. In experiment two however, the flashes could be presented 10° either to the right or left side of the fixation cross. The beeps were presented from two small speakers, each placed immediately below the screen at the eccentricity that the flashes were to appear. The 20° separation thus obtained is comfortably larger than the minimum discernable angles for auditory [28] and visual stimuli. Although simple sinusoid stimuli were used that may be hard to locate due to their reduced spectral complexity, they were presented in free-field conditions designed to maximise ease of localization.

### Procedure

In the first experiment, participants sat in a chair with their eyes at a distance of 70 cm from a computer screen. The fixation cross was displayed alone for an interval that varied randomly on each trial between 1200 and 1500 ms. The flash/beep sequence then began. Following the sequence was another short randomly varied interval (1200 to 1500 ms), after which the text “How many flashes did you see?” was displayed in place of the fixation cross. This text remained in place until the participant made a response on the keyboard. Participants were instructed to keep their gaze on the fixation cross during each trial and count the number of flashes that would appear whilst ignoring the beeping sounds. The response was made after each trial by pressing keys numbered one to four on a keyboard. Each of the eleven possible beep/flash combinations (1F0B, 1F1B, 1F2B, 1F3B, 1F4B, 2F0B, 2F1B, 3F0B, 3F1B, 4F0B, 4F1B) was presented randomly in a single block. This block was repeated fives times (with trials re-randomised each time).

In the second experiment, the sources of the auditory and visual stimuli were separated. As in experiment one, during one-flash trials zero to four beeps were presented. On multi-flash trials, either zero or one beep was presented. There were four possible spatial configurations: two congruent configurations where the beep and flash are presented together on the left and together on the right (termed LL and RR), and two incongruent configurations where flashes on the right are paired with beeps on the left, and vice versa (termed LR and RL). On the seven trials when both beeps and flashes were present (1F1B, 1F2B, 1F3B, 1F4B, 2F1B, 3F1B, 4F1B), all four spatial configurations were displayed (LL, LR, RL and RR), and for those with only flashes present (1F0B, 2F0B, 3F0B, 4F0B), only two configurations were possible (LL and RR). There were thus 36 types of trials. The experiment was broken into five blocks, in which each trial type was presented five times. Within each block trials were ordered randomly.

## Results

### Central presentation - mean responses

**Figure 2:**
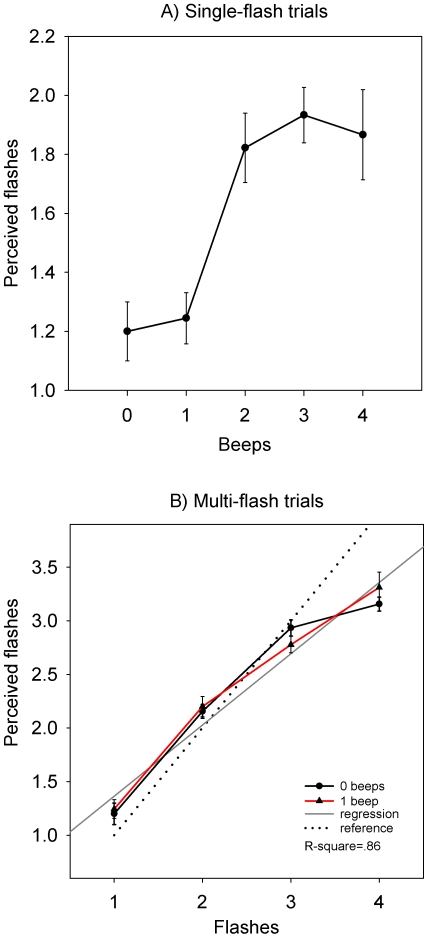
Experiment 1 mean responses. Mean responses for the number of flashes reported (bars show SEM) for all trial types in experiment one. Single-flash trials are shown in panel A, and multi-flash trials in panel B. The reference line in panel B shows veridical perception.

Fig. 2A shows the mean responses averaged across the nine participants (error bars show standard error of the mean) when a single flash was presented. Increasing the number of accompanying beeps dramatically increased the number of flashes reported when only one flash was present. The increase was strongest in the case where one flash was accompanied by two beeps compared to a single beep. Increasing the number of accompanying beeps to three increased the average response further, but the effects were not significant. In order to test the effect of beeps on the number of flashes reported, the mean responses for the five 1-flash trials were submitted to a repeated-measures Analysis of Variance (ANOVA), with a 5-level within-subjects factor Beep (0–4 beeps). The main effect of Beep was significant (*F*(4,32) = 17.5, *p<*0.01). When a single flash was presented the number of beeps had an effect on the number of flashes reported. Post-hoc comparisons revealed a significant increase in the mean number of flashes reported from 1.2 (*SD* = 0.3) when one beep was presented to 1.8 (*SD* = 0.2, *p* = 0.001) when two beeps were presented (Fig. 2A).

Fig. 2B shows trials where more than one flash was presented, with separate lines for trials with zero and one beep. It can be seen that there is very little difference between trials with or without beeps, indicating that the presence of a single beep did not affect the ability of the participants to correctly count any number of flashes. To test whether responses increased as the number of flashes presented increased, and whether this relationship changed depending on whether beeps were presented concurrently, the mean responses were submitted to a repeated measures ANOVA. There were two within-subjects factors – Flashes (1, 2, 3 or 4) and Beeps (0 or 1). There was a significant main effect of Flashes (*F*(3,24) = 176.8, *p*<0.001) but no significant effect of the number of beeps. Post-hoc tests indicated that the number of flashes reported increased significantly between each adjacent level of flashes presented.

The reference line in Fig. 2B indicates veridical perception – the responses that could be expected if flash perception were perfectly accurate. It can be seen that for one to three flashes, the measured responses (whether a beep was presented concurrently or not) are close to the reference line, indicating that for up to three flashes the stimuli are relatively easy to perceive. For four flashes however, participants consistently under-report the number of flashes presented (*M* = 3.2, *SD* = .1). Further analysis will therefore exclude the four-flash stimuli on the basis that they are ambiguous.

After excluding the four-flash stimulus, a linear regression analysis showed that the number of flashes reported was a highly significant predictor of the number of flashes actually presented (*R^2^* = 0.86, *F*(1,52) = 367.4, *p*<0.001), with a nearly unitary slope (*β* = 0.93, *t*(52) = 19.2, *p*<0.001), indicating that as the number of flashes presented increased by one, the number flashes reported was also likely to increase by one.

### Central presentation - response rates

**Figure 3:**
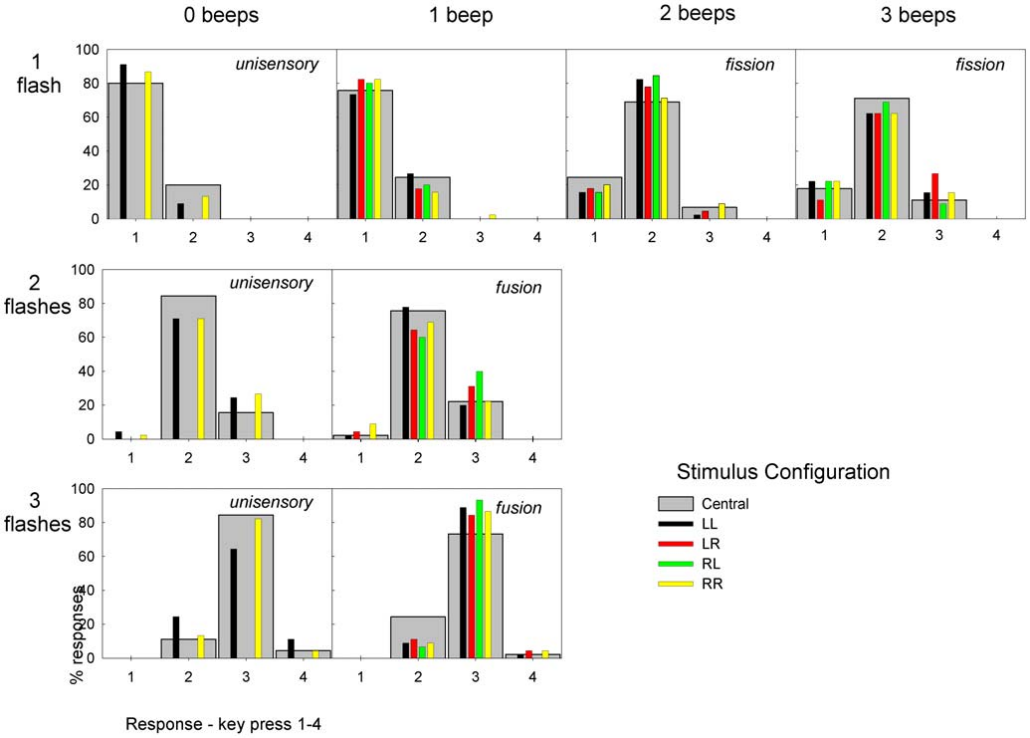
Mean response rates (%). Means response rates (%) are shown for both experiment one (grey bars) and experiment 2 (coloured bars). Unisensory, fission- and fusion-capable stimuli are marked with italic text.

Following [17], the stimuli were divided into three classes – those capable of producing fission illusions (1F2B, 1F3B), those capable of producing fusion illusions (2F1B, 3F1B) and the unimodal visual stimuli (1F0B, 2F0B, 3F0B). A categorical analysis was then performed on the response counts for fission and fusion stimuli. Mean response counts (expressed as a percentage of the total number of trials) for each stimulus type are shown in Figure 3 (wide grey bars only).

Counts of fission responses were calculated by summing the number of responses indicating more flashes than were presented for the two fission-capable stimuli. Similarly, non-fission counts were computed by summing responses indicating the correct number or fewer flashes than were presented. The ratio fissions:non-fissions then indicated the odds of a fission illusion occurring with a multisensory stimulus. For both fission stimuli the odds of fission with the equivalent unisensory stimulus (1F0B for both fission stimuli) was similarly determined. Finally, the odds ratio of fission in the multisensory case vs. the unisensory equivalent was calculated. A value of greater than one in this final odds ratio thus indicates a greater likelihood of fissions for the audiovisual stimulus compared to its unimodal equivalent. The significance of the association between the stimulus type (uni vs. multi-sensory) and the report of illusions (fission/fusion vs. non-fission/non-fusion) was assessed with a Chi-square test. To follow [17], the significance of the association was also tested using Fisher's exact (one-sided) test, although it should be noted that two out of the four cells in the fusion Chi-square test have expected counts of less than five, violating the assumptions for that test. The results of both these tests, along with the corresponding odds ratios, are shown in Table 1. Both fission stimuli showed a significant association between the type of stimulus (uni- or multi-sensory) and the report of an illusion, with both multisensory stimuli approximately 30 times more likely to elicit an illusion response than unisensory stimuli. Neither fusion stimulus showed a significant association.

**Table 1:** Odds for fusion and fission illusions, Experiment 1 (central presentation).

Stimulus	Odds of illusion (multisensory)	Odds of illusion (unisensory)	Odds ratio	*X* ^2^(df)	*X* ^2^ *p*	Fisher *p* (one-sided)
1F2B (fission)	3.1	.25	12.4	27.8(1)	<.000	<.000
1F3B (fission)	4.6	.25	18.5	34.9(1)	<.000	<.000
2F1B (fusion)	.02	.02	1.0	1.0(1)	.3	.5
3F1B (fusion)	.03	.02	2.6	2.7(1)	.09	.08

The odds for fusion and fission illusions for each of the four illusion-capable stimuli in Experiment One (central presentation). The final odds ratio, and the significance of the association between stimulus type (uni- vs. multi-sensory) and reports of illusions (fissions/fusions vs non-illusory) are also shown.

### Spatial presentation - mean responses

**Figure 4:**
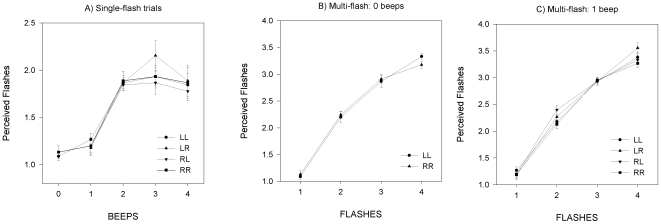
Experiment two mean responses. Mean responses for the number of flashes reported (bars show SEM) for all trial types in experiment two. Single-flash trials are shown in panel A. Multi-flash trials with zero beeps and one beep are shown in panels B and C respectively.

As in experiment one, mean responses were calculated for each trial type, and are displayed in Fig. 4a. To determine the effect of the number of beeps in the four different spatial configurations on the number of flashes reported, the mean responses for the sixteen stimulus types in which both beeps and flashes were present were submitted to a 4 (Beeps: 1, 2, 3, 4)×4 (Stimside: LL, LR, RL, RR) repeated measures ANOVA. There was again an effect of Beeps (*F*(3,24) = 34.0, *p*<0.001), but no significant effect of Stimside, and no interaction between the number of beeps and the Stimside condition. As in experiment one, contrasts revealed that the number of flashes reported increased significantly when the number of beeps presented increased from one to two (*F*(1,8) = 114, *p*<0.001).

To investigate whether participants showed any spatial bias towards either the left or right in their ability to count the flashes in the absence of any auditory stimulus, the mean responses when no beeps were present were examined (Fig. 4B). For the eight stimulus types in which flashes were presented alone, mean responses were submitted to a 2 (Stimside: LL, RR)×4 (Flashes: 1, 2, 3, and 4) repeated measures ANOVA. There was a significant main effect of the number of flashes presented (*F*(3,24) = 298, *p*<0.001), but no effect of the side that the flash was presented (*F*(1,8) = 0.001, *p* = 0.9).

Next, the single-beep trials were analysed (Fig. 4C). Analysis of these trials allowed examination firstly of whether separation of the auditory and visual components of the stimulus affected the participants ability to count flashes overall (similarly to the zero-beep trials), and secondly of whether there might be a change in this ability depending on the exact stimulus configuration. Mean responses for single-beep trials were submitted to a 4 (Flashes: 1, 2, 3, and 4)×4 (Stimside: LL, LR, RL, RR) repeated-measures ANOVA. There was a main effect of the number of flashes (*F*(3,24) = 436, *p*<0.001), but no main effect of the stimulus configuration, nor an interaction between the stimulus configuration and the number of flashes.

### Spatial Presentation - Response rates

As the stimulus configuration was found to have no effect on responses to the flash stimulus in illusion conditions or otherwise, the stimulus configuration factor was collapsed and the data from experiments one and two were pooled. However, as trials with both flashes and beeps had four possible spatial configurations compared with the two possible with flash-only trials, only the LL and RR trials (common to all trial types) were added. The response rates using this larger number of trials were then re-analysed. To enable direct comparison with results from [17], the odds ratio calculated for the sums of all fission stimuli and all fusion stimuli were computed. The results are shown in Table 2. The pattern of results is similar to those from Experiment 1. Both fission stimuli show significant associations between the stimulus type and the odds of an illusion response, while both fusion stimuli show no significant association. For the summed fusion and fission stimuli the pattern is again the same – the association between stimulus type and the odds of an illusion response is significant for fission stimuli, but not fusion stimuli.

**Table 2:** Odds for fusion and fission illusions, Experiment 2 (spatial presentation).

Stimulus	Odds of illusion (multisensory)	Odds of illusion (unisensory)	Odds ratio	*X* ^2^(df)	*X* ^2^ *p*	Fisher *p* (one-sided)
1F2B (fission)	4	.16	24.4	117.8(1)	<.001	<.001
1F3B (fission)	3.8	.16	22.34	115.2(1)	<.001	<.001
2F1B (fusion)	.04	.02	2.0	1.0(1)	.3	.5
3F1B (fusion)	.16	.19	.8	.26(1)	.6	.7
All fission	3.9	.10	38.3	233.0(1)	<.001	<.001
All fusion	.10	.10	1	0(1)	1.0	1.0

The odds of fusion and fission illusions for multisensory stimuli and their uni-sensory equivalents in combined data from experiments 1 and 2. The LL and RR spatial configurations were collapsed and pooled with the data from experiment one. The rows labelled “All fission” and “All fusion” show data summed across all fission- and fusion-capable stimuli, respectively. The final odds ratio, and the significance of the association between stimulus type (uni- vs. multi-sensory) and reports of illusions (fissions/fusions vs non-illusory) are also shown.

## Discussion

The experiments reported here replicate the flash-beep fission illusion described by Shams *et al.* [6], [23], however we find no evidence for an associated flash-beep fusion illusion reported in later studies [9], [12], [17], [29]. We also show that the flash-beep illusion is not affected by spatially separating the auditory and visual components of the stimulus. These results suggest that although the illusion has been previously shown to be compatible with the temporal rule describing neural activation in the SC, it is not bound by the corresponding spatial rule. The illusion thus may have neural origins that are outside the regions governed by these rules.

The results in experiment one were similar to those obtained by Shams *et al* [23] – when two or more beeps were presented along with a single flash, participants reported seeing two or more flashes. The number of flashes reported increased from approximately one, to approximately two when the number of beeps presented increased from one to two. As in the Shams *et al* study, the number of flashes reported did not increase significantly beyond two as the number of beeps increased further to three and four.

The results in the control trials also echoed those in the Shams *et al.* study. In zero-beep trials, it was found that participants were able to count up to three flashes relatively easily, indicating that the visual stimulus when presented alone was unambiguous up to that point. However, in the Shams *et al.* study the participants appear to be more accurate with four flashes than in the present study (compare their Fig. 4A with our Fig. 2B). In the present study, there were no differences in the number of flashes reported between zero-beep and one-beep trials. This held for any number of flashes presented, and indicates that the presence of an auditory stimulus per se does not appear to affect the number of flashes reported. After excluding the four-flash trials and pooling the zero-beep and one-beep trials, the number of flashes presented was a significant predictor of the number of flashes reported, again indicating that our participants had no difficulty reporting the number of flashes presented. In summary, with the exception of the four-flash stimuli, the results in the first experiment closely followed those reported by Shams *et al.* [23], suggesting that any differences in stimuli, participants or procedure did not lead to a reduction in the strength of the illusion.

In their study, Shams *et al*. [23] found that an illusion occurred only when the number of beeps exceeded the number of flashes and not vice versa. The well established “modality appropriateness” hypothesis [30] thus could not explain their results. Audition provides more accurate temporal information than vision, and thus should be the more appropriate modality for this task. According to this theory, the number of beeps should thus always dominate over the number of flashes. However, in the results from Shams *et al.* [23], this did not appear to be the case, and the authors proposed instead the “discontinuity hypothesis” – that the discontinuous stimulus in one modality alters the percept of the continuous stimulus in the other modality. The present results fit with this pattern, thus also suggesting that the modality appropriateness hypothesis does not fully explain the flash-beep illusion.

In addition to the sound-induced flash (or fission) illusion, Andersen *et al.* [17] were the first to report a corresponding sound-induced fusion illusion. In their study, participants reported seeing fewer flashes than were actually presented on trials with fewer beeps than flashes. This fusion effect was weaker than the fission illusion, and disappeared when the auditory stimulus was reduced to a near-threshold level. Rather than supporting one theory or the other, the authors suggested that modality appropriateness and discontinuity are both factors that combine to influence the dominance of each modality. Several other studies have now shown similar results [12], [29], [31], with participants reporting fewer flashes than were presented in trials with fewer beeps than flashes. In order to compare the present results with Andersen *et al.* [17] our data were re-analysed following their categorical analysis technique. The results are strikingly different – firstly the likelihood of fission illusions was almost five times higher in the present data (see Table 2), and secondly no evidence was found for fusion illusions occurring.

There are several possibilities for the discrepancy in these results. Firstly, in the present study the stimulus timing in Shams *et al.* [23] was followed as closely as possible, with the auditory stimulus leading the visual by 23 ms (when both auditory and visual stimuli were present). Andersen *et al.* [17] used a simultaneous presentation of the flash and beep. It is possible that by presenting the auditory stimulus 23 ms before the flash, multiple flashes were somehow rendered less likely to fuse than if they were presented simultaneously. In other respects the stimuli are closely matched. Other studies that have reported a fusion illusion in the 2F1B condition [9], [12] have also presented the auditory and visual stimuli simultaneously. However one study using simultaneous auditory-visual stimuli [32] did not find any evidence of fusion, and another [29] used non-simultaneous auditory and visual stimuli, and did find auditory-visual fusion. The only study to directly investigate the effect of stimulus timing on the illusion [23] found that varying the auditory-visual onset by ±70 ms had little effect on the strength of the fission illusion, but with separation beyond 70 ms the strength of the illusion gradually declined. However, only the fission-illusion stimuli were included in their investigation. Stimulus timing may have a different effect on different stimulus combinations.

Another influence on stimulus timing is the nature of the display system itself. The flash stimulus is typically quoted as being of 17 ms duration, with a flash onset asynchrony of 67 ms (with the exceptions of Meylan & Murray [32] – duration 13 ms, onset asynchrony 65 ms; Mishra et al [9] – duration 5 ms, onset asynchrony 70 ms, and Shams *et al.* [29] – duration 10 ms, onset asynchrony 70 ms). The flash durations reported in the literature generally correspond to the refresh period of the display (usually set at 60 Hz, giving an intended “flash duration” of 16.7 ms) rather than the measured duration of the flash. However, different display types can have widely varying decay times, so that a single refresh of a liquid crystal display (LCD) monitor or projector for instance may produce a flash stimulus substantially longer than that produced by a CRT monitor, despite the refresh rates and intended flash durations being identical. In the present study, the exact duration of a single-refresh flash of the CRT set to a refresh rate of 60 Hz was measured using a photodiode and oscilloscope at 1.3 ms (FWHM), rather than the 17 ms that would be quoted by assuming that the flash duration is equal to the refresh rate period. An examination of a variety of other CRT and LCD monitors revealed similar flash durations among CRT monitors (1.2–1.5 ms FWHM), but much longer flash durations for LCD screens (12 ms FWHM). A longer flash stimulus would result in a shorter inter-flash interval in multi-flash trials, and may render two consecutive flash stimuli more likely to fuse. Of the four studies finding evidence for a fusion illusion, two have used LCD projectors [11], [12], made necessary as the experiments were performed in a functional magnetic resonance imaging scanner. A light-emitting diode was used in another [9], and [17] and [29] do not report the display used.

Secondly, while Andersen *et al*. [17] used all twelve possible combinations of stimuli (0–3 beeps and 1–3 flashes), a reduced set of eight stimuli was used in the present study. As a result, counts for the frequency of fission and fusion illusions were pooled across two stimulus types (fissions: 1F2B+1F3B, fusions: 2F1B+3F1B) rather than the three used in [17]. For fusion stimuli in particular this may have had a large effect. In [17], only 50% of responses to the three-flash stimulus were correct even in the absence of any auditory stimulus. It is therefore difficult to interpret any further effects of the auditory stimuli in that study. In the present study, participants were more accurate with the 3F0B (80% correct) and 3F1B (75% correct) stimuli (Fig. 3), again possibly due to differences in the display characteristics. With the visual stimulus more easily perceived, it might be that any fusing effect of the auditory stimulus was rendered less effective in the present study.

After validating the procedure, the effect of spatially separating the auditory and visual components of the multisensory stimuli in the illusion was examined. The flash-beep illusion can so far be understood in terms of only one of the three multisensory rules – the temporal rule. The illusion is strong with temporal variability up to around 70 ms, after which point it gradually disappears [23]. This figure ties in closely with temporal integration times for multisensory neurons in the superior colliculus [21]. Shams *et al*. [23] theorize that the illusion is the result of auditory processing modifying visual perception rather than a result of decision-making biases. If true, it is thus likely to at least in part be supported by functions of the SC, and is already known to obey the temporal rule. However, the present results indicate that despite separating the stimuli in space to an extent that should be easily perceived, no aspect of performance on the task changed, both on illusion and control trials.

There have now been many electrophysiological studies in cats and monkeys [20], [33] indicating the existence of neurons in the SC that respond in an integrative manner to multisensory stimuli. These neurons are sensitive to temporal and spatial congruence between the auditory and visual signals from a multisensory stimulus – as the two signals are moved further apart in time or space, the responses from these multisensory neurons reduce. This phenomenon has been echoed at a behavioural level in humans, with performance on multisensory tasks exhibiting similar spatial [22] and temporal [1] response gradients. In [1], participants were asked to fixate centrally and detect sub-threshold masked flash stimuli displayed at 8, 24, 40 and 56 degrees in the left and right visual fields. The task was performed in a vision-only condition, as well as an auditory-visual condition where sounds were presented either at the same or different locations to the flash. They found that perceptual sensitivity generally improved when an auditory signal was presented at the same location. Interestingly, there was one exception to this finding: when an auditory stimulus was presented 16° further to the right of the visual stimulus at 40°, perceptual sensitivity was enhanced. When the sound was played at other locations there was no improvement, despite adjacent locations being only 16° apart. These results are thought to reflect the fact that performance on such tasks is closely related to multisensory processes in the SC. In a follow-up study [34], a simple audio-visual detection task was administered using red or blue/purple visual stimuli. Blue/purple stimuli are detected using only S-type cones in the retina, which do not project directly to the SC [35], [36]. Reaction time measures showed evidence for multisensory integration only using the red stimuli (which do project to the SC), providing evidence for the involvement of the SC in audiovisual multisensory integration in humans. Furthermore, the reaction time effect for red stimuli diminished when the sources of the auditory and visual stimuli were separated, and when a 250 ms delay was added between the auditory and visual stimuli. In comparison, although the sound-induced flash illusion has been shown to be consistent with the temporal rule, in the present experiment separating the spatial origins of the visual and auditory stimulus by 20° did not change reports of the illusion in any detectable way. The results suggest that the rules of multisensory integration as they apply to neurons in the SC may not hold in the case of the flash-beep illusion.

While most studies find that the facilitatory effect of multisensory stimulation requires that the two stimuli be spatially coincident [1], [37], [38], this does not always seem to be the case. In a series of three experiments Stein and colleagues [39] found that during a luminance intensity judgment task, participants reported higher intensities on trials in which there was an accompanying irrelevant auditory stimulus, even when the sound was located at a random position up to 45 deg away from the visual stimulus. However, when both the auditory and visual stimuli were moved away from fixation, luminance judgements were no longer enhanced. This result is contrary to many that fit with the spatial rule describing properties of multisensory neurons in the SC [40], [41] as well as behaviourally in humans [22], [42], [43]. The authors note that although the spatial rule is well established at the level of individual neurons in the SC, there are multisensory neurons located in many other areas of the brain involved in many different tasks for which stimulus localisation in space is possibly not vital. They suggest that the task of assessing stimulus intensity could be one such activity. The results from the current study follow a similar pattern – behaviour related to the illusory phenomenon seems to echo known temporal rules for multisensory integration in the SC, but not spatial rules.

If the flash-beep illusion is indeed a case of a visual percept induced by an auditory stimulus, the question remains as to how in the brain this might occur, particularly in the timeframe available. It is becoming increasingly recognised that multisensory integration can take place not only via the SC and other traditionally multisensory areas of the brain, but also directly between primary sensory cortices (see [24], [44] for reviews). This view is in agreement with findings indicating that sensory-specific judgements (such as contrast for vision) can be affected by information from another sense. In the context of the sound-induced flash illusion, this has recently been seen in a study indicating that the illusory flash has a detectable contrast [19]. Using event-related potentials, it has also been shown that while late (more than 100 ms) multisensory effects on visual processing are sensitive to the spatial congruence of the stimuli [45], the earliest (∼50 ms) multisensory effects generally occur irrespective of the stimulus location [27], but are sensitive to temporal congruence [46]. The results from the present study generally fit with this view – in order for the auditory stimulus to affect visual perception, integration of the auditory information would have to occur very rapidly, perhaps via direct cortical pathways that are not sensitive to spatial information.

It also worth pointing out that retrograde tracing studies in primates have found that the inputs from the core and parabelt auditory cortex to primary visual cortex provide their strongest connections to areas of primary visual cortex that subserve the peripheral visual field – from 10–20° eccentricity [25]. The sound-induced flash illusion is strongest when the flash is presented in the periphery, and Shams *et al* [7] have shown that illusion-related modulations of the flash VEP occur only when the flashes are presented in the visual periphery. Generally an eccentricity of 5–20° has been used in studies of the illusion, with most studies presenting stimuli within the range where connections from primary auditory to primary visual cortex have been found subserving the visual periphery [25]. Although the minimum audible angle for sounds is only a few degrees, spatial receptive fields of individual neurons in the auditory cortex are large, generally occupying a quadrant or more of acoustic space with stimuli 10–30 dB above neural thresholds [47]. For a recent explanation of how individually broad receptive fields are thought to translate to high spatial accuracy, see [48]. With higher sound levels, the receptive fields broaden still further [49], despite higher-intensity sounds being easier to localise [50]. These direct cortico-cortical connections from neurons in the auditory cortex with very wide spatial receptive fields to neurons subserving the periphery in primary visual cortex is an alternative cortical mechanism that may underlie the illusion, and its insensitivity to spatial incongruence.

In summary, the present results firstly replicate the sound-induced fission illusion described by Shams *et al*. [6], [23]. In our replication however we do not find an associated flash-beep fusion illusion [17], and speculate that the variety of findings in the literature concerning the fusion illusion may be due to unintended differences in stimulus timings brought about by different visual displays. Secondly, the present results show that the illusion is insensitive to spatial incongruence of the auditory and visual stimuli. Although the design of the present study cannot directly address the nature of the underlying neural mechanisms, the pattern of results are consistent with recent research [24], [27], [46] showing that direct cortical connections between primary sensory areas [25] may be sensitive to temporal, but not spatial congruence.
